# Supplementary material for: On-surface synthesis of phthalocyanines with extended π-electron systems
Source: Commun Chem. 2024 Dec 9;7:292. doi: 10.1038/s42004-024-01351-8 (PMC11628558; doi:10.1038/s42004-024-01351-8)
Supplement: Supplementary file 2 — Supplemental materials [file 42004_2024_1351_MOESM2_ESM.pdf]

Supplementary Information

On-Surface Synthesis of Phthalocyanines with Extended π-Electron Systems

Lukas J. Heuplick[1], Qitang Fan[1,2,3], Dmitriy A. Astvatsaturov[4], Tatiana V. Dubinina[4], J. Michael Gottfried[1*]

[1]*Department of Chemistry, University of Marburg, Hans-Meerwein-Str. 4, 35032 Marburg, Germany, E-Mail: gottfried@uni-marburg.de*

[2]*Hefei National Research Center for Physical Sciences at the Microscale and Synergetic Innovation Center of Quantum Information & Quantum Physics, New Cornerstone Science Laboratory, University of Science and Technology of China, Hefei, Anhui 230026, China*

[3]*Hefei National Laboratory, University of Science and Technology of China, Hefei, Anhui 230088, China*

[4]*Department of Chemistry, Lomonosov Moscow State University, 119991 Moscow, Russian Federation*

Table of Contents

1. Supplementary Methods
    1.1. Synthesis of Precursors
2. Supplementary Figures
    2.1. Further Data on NND/Ag(111): Cyclodehydrogenation at 630 K and Experiments with Co-Adsorbed Iron
    2.2. Detailed Analysis of Conformer Contributions
    2.3. Changes to the Reaction due to Increased Fe-to-Molecule Ratios
    2.4. Further Data on the Polycyanine and Ag-NPc Formation of PND
    2.5. Additional C 1s XPS Spectra
    2.6. Desorption of PND from the Au(111) Surface
    2.7. Reactivity of NND on Au(111) and Formation of Au-NPc
    2.8. Further STM data of PND on Au(111)
    2.9. Determination of the Crystal Orientation
    2.10. Size Adjustments of Molecular Overlays

1. Supplementary Methods

1.1. Synthesis of Precursors

1.1.1. Preparation of 6,7-diphenyl-2,3-naphthalenedicarbonitrile

A mixture of 6,7-dibromonaphthalene-2,3-dicarbonitrile (1.80 g, 5.36 mmol), phenylboronic acid (2.28 g, 18.70 mmol) and a saturated aqueous solution of K_2CO_3 (5 mL) were stirred in 96 mL of boiling mixture 1,4-dioxane:acetonitrile (8:3 V/V) under argon. The dichloro-bis(triphenylphosphine) palladium compound (0.038 g, 0.054 mmol) was added after boiling the solvent. The reaction was carried out for 6 h (TLC-control: Al_2O_3, C_6H_6). The reaction mixture was cooled to room temperature and water was added. The product was collected by extraction with ethyl acetate. The residue was treated by flash chromatography (ethyl acetate:hexane (1:2)) in order to remove the catalyst destruction products. The resulting compound was additionally purified by chromatography on a column with Al_2O_3 using a mixture of ethyl acetate:hexane (1:2) as the eluent to give pale yellow crystals of PND (1.5 g, 85%).

1.1.2. Preparation of 6,7-di(2-naphthyl)-2,3-naphthalenedicarbonitrile

A mixture of 6,7-dibromonaphthalene-2,3-dicarbonitrile (0.5 g, 1.49 mmol), 2-naphthylboronic acid (1.54 g, 8.95 mmol) and a saturated aqueous solution of K_2CO_3 (1.23 g, 8.95 mmol) were stirred in 29 mL of boiling mixture 1,4-dioxane:acetonitrile (2.6:1, V:V) under argon. The dichloro-*bis*(triphenylphosphine) palladium compound (0.02 g, 0.029 mmol) was added after boiling the solvent (b.p.=84°C, 760 mm Hg). The reaction was carried out for 4 h (TLC-control: Al_2O_3, ethyl acetate:*n*-hexane, 1:10, V:V). The reaction mixture was cooled to room temperature and 150 mL of water was added. The product was collected by extraction with ethyl acetate and dried with $CaCl_2$. The residue was purified by gradient chromatography using benzene and then ethyl acetate as the eluent. The resulting compound (fraction moved by ethyl acetate) was dissolved by benzene and then *n*-hexane was added. The resulting precipitate was dried at room temperature, yielding target compound NND (0.41 g, 64%). R_f =0.43 (ethyl acetate:*n*-hexane, 1:10, V:V). ^1H NMR δ_H (400.13 MHz, [D_6]DMSO) 7.16-7.19 (2H, dd, *J*=8.5 Hz, 1.7 Hz); 7.51 (2H, d, *J*=3.3 Hz); 7.52 (2H, d, *J*=3.3 Hz); 7.69 (2H, d, *J*=8.5 Hz); 7.83-7.85 (2H, dd, J=6.1, 3.5 Hz); 7.86-7.88 (2H, dd, J=6.1, 3.5 Hz); 8.02 (2H, s); 8.41 (2H, s); 8.96 (2H, s). ^{13}C NMR δ_{13} (150.90 MHz, [D_6]DMSO) 108.97; 116.47 (CN); 126.47; 126.51; 127.34; 127.38; 127.46; 127.97; 128.40; 130.61; 131.89; 132.20; 132.74; 136.37; 137.18; 143.13. IR ν (KBr): 2231 cm^{-1} (st CN). MS-EI (I/Imax, %) m/z: 430 [M, 100%]; calculated for $C_{32}H_{18}N_2$ 430.1470, found for [M] 430.1470.

2. Supplementary Figures

2.1. Further Data on NND/Ag(111): Cyclodehydrogenation at 630 K and Experiments with Co-Adsorbed Iron

Further annealing of the NND-based naphthalocyanine molecules on Ag(111), as shown Figure 2e, above 500 K is expected to induce intramolecular cyclodehydrogenation of the naphthyl substituents, resulting in flattening of the ligand. Indeed, after annealing to 630 K we no longer see molecules with regular bright protrusions at the periphery. In addition, all ordered structures disappeared (Figure S1a). The close-up image in Figure S1b confirms the presence cyclic tetramers with lobes of uniform brightness and a central protrusion. We attribute these structures to the flattened Ag-NPc in the Ag-up configuration. Besides the intramolecular reaction, also intermolecular dehydrogenative C-C coupling occurred, as can be seen in Figure S1b, which shows molecules that are linked at the periphery. These covalent aggregates can easily be imaged due to their low mobility, while the additional fuzzy features in Figure S1a,b suggest the presence of highly mobile molecules, which may be flat Ag-NPc molecules that did not undergo intermolecular coupling.

To get further understanding about the differences between NDD and the smaller PND, we prepared an additional NND sample with co-adsorbed iron atoms. Figure S1c show the resulting structure after deposition at 300 K. Upon annealing to 500 K cyclic oligomers of irregular appearance are formed (Figure S1d). This result contrasts the regular cyclic tetramers (Fe naphthalocyanines) that were obtained by reaction of PND and Fe (cf. Figure 3 and 6).

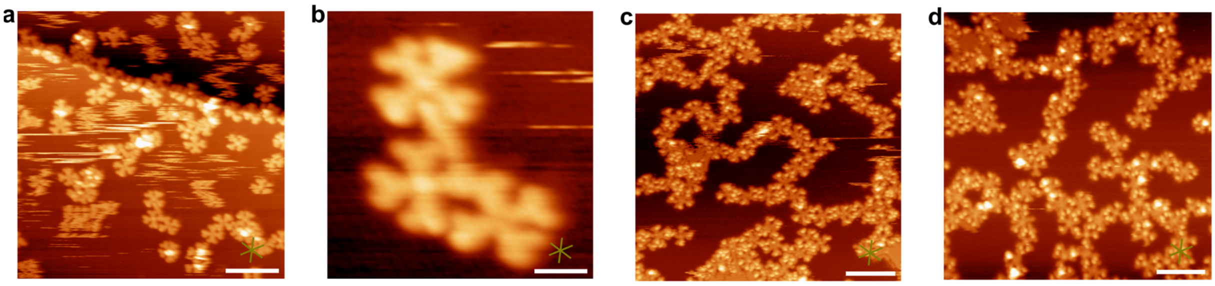

Figure S1. (a, b) Overview and close-up STM images of NND/Ag(111) upon annealing to 630 K. (c, d) STM images of NND/Ag(111) in presence of co-adsorbed iron after deposition at 300 K and annealing to 500 K, respectively. Scale bars: (a) 10 nm; (b) 2 nm; (c, d) 8 nm. Tunneling parameters: (a) U = -2.59 V, I = -0.11 nA; (b) U = -2.59 V, I = -0.16 nA; (c) U = -1.74 V, I = -0.14 nA; (d) U = -2.15 V, I = -0.10 nA.

2.2. Detailed Analysis of Conformer Contributions

In the two-dimensional (2D) confinement on the surface, PND can exhibit one conformer and NND can exhibit four conformers, if only the relative orientation of the aryl substituents relative to each other is considered. However, due to the intrinsic tilt of the substituents, there are two cases for each conformer, depending on whether the right or the left substituent is tilted. In the case of the intact PND precursor upon deposition at 300 K, the only island that could be resolved with sufficient detail (Figure 3b) shows exclusively the “right side up” conformer. However, in absence of another image with sufficient sub-molecular resolution, we cannot prove or exclude that a corresponding mirror domain exists. Upon annealing PND in the presence of Fe to 500 K, we observed two different islands (Figure S2a and S2b) and can find at least 3 different Fe-NPc products, as illustrated in Figure S2c. Apparently, both PND conformers appear in the reacted Fe-NPc, with no clear preference for one of them.

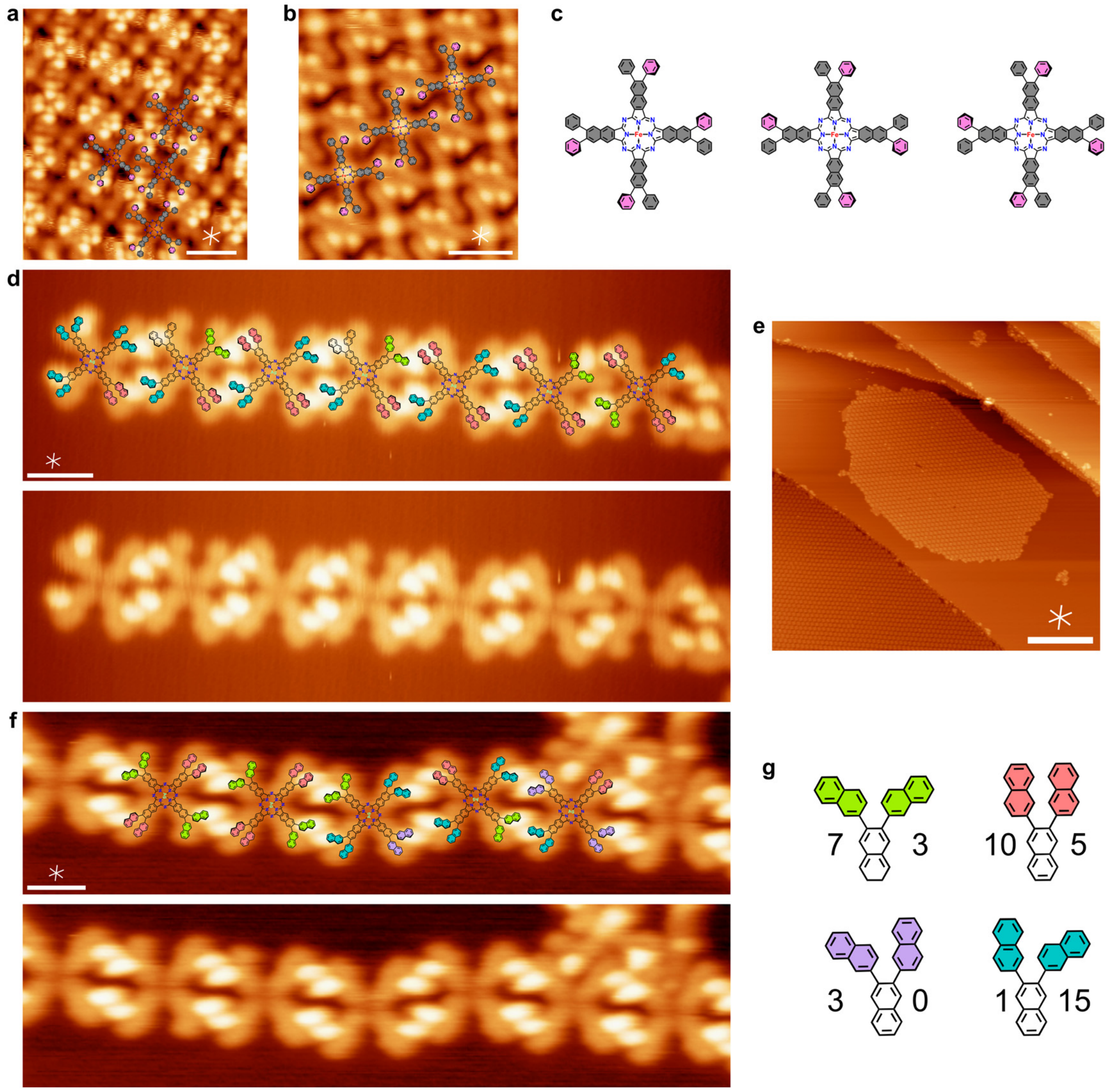

Figure S2. (a,b) Close-up STM images of an island of PND-derived Fe-NPcs at a slightly different crystal positions, cf. also Figure 3e. (c) Observed conformers of Fe-NPc taking the tilt of the phenyl rings into account. (d, f) Two different NND-derived Ag-NPc ribbons at different crystal positions with and without molecular overlay. (In Figure 2g in the manuscript, a fraction of Figure S2d was overlaid with chemical models.) (e) Overview image of the NND precursor before annealing, demonstrating the presence of more than one island. (g) Contributions of the four different NND conformers in Figure S2d,f. The numbers give the observed number of conformers considering the tilting of the left or right naphthyl substituent. Scale bars: (a, b, d, f) 2 nm; (e) 40 nm. Tunneling parameters: (a) U = -1.00 V, I = -0.23 nA; (b) U = -2.59 V, I = -0.14 nA; (d) U = -2.75 V, I = -0.10 nA; (f) U = -2.75 V, I = -0.08 nA.

For NND, the above consideration leads to 8 different conformations of the monomer units and therefore to manifold combinations in the Ag-NPc chains (Figure S2d and S2f). For the intact NND precursor in Figure 2b in the manuscript, we were able to identify three different conformers in one noncovalent tetramer. Possibly, there are noncovalent tetramers which are composed of different conformers, but due to the lack of high-resolution data for more islands, this cannot be confirmed with certainty. However, already the overview image in Figure S2e suggests that not all assemblies might be equivalent. The center terrace shows a large island that is isolated from the step edges, while another island (bottom-left) extends up to the step edge. The two islands appear to be mirror domains, but are additionally rotated by an angle of ~10° relative to each other. However, we have no insight regarding the distribution of conformers in the two islands, or whether one conformation is more stable, or whether the conversion barrier is low enough for a statistical random distribution at room temperature.

Analyzing two different chains of naphthalocyanines obtained by annealing of NND, we find that there is not a singular most common monomer combination in the Ag-NPcs. By individually counting all four conformers, we find an approximately statistical ratio. The mixed (blue and purple) conformer occurs 19 times, the V-shaped (green) conformer occurs 10 times, and the parallel conformer (red) occurs 15 times. However, further considering the tilted side (cf. the numbers in Figure S2g) there seems to be a trend for a specific side in each conformer, which may be due to intermolecular and molecule-substrate interactions.

2.3. Changes to the Reaction due to Increased Fe-to-Molecule Ratios

In both PND/Fe samples shown in Figure 3 (on Ag(111)) and Figure 6 (on Au(111)), iron-to-molecule ratios below 1 Fe per 4 precursor molecules were discussed. This led to the desorption of excess PND molecules upon annealing, resulting in the stoichiometric ratios (1 Fe atom per 1 NPc molecule formed). Figure S3 shows results for samples with increased initial Fe-to-molecule ratios. On Ag(111), the ratios to up to over-stoichiometric amounts of Fe (Figure S3a,b after deposition at 300 K; Figure S3c,d after annealing to 500 K), while on Au(111), the ratios remain in the sub-stoichiometric regime (Figure S3e,f). As can be seen, the high Fe coverage on Ag(111) leads to a completely changed reaction behavior. Instead of regular fourfold symmetric features, various irregular flower-shaped structures are obtained. Possibly, these are higher cyclic oligomers comprising five and more monomer units, with a cluster of Fe atoms in the center. Based on the available data, however, it is not possible to clarify the structure unambiguously.

On Au(111), increasing the amount of Fe to ratios still below the stochiometric does not seem to affect the formation of the regular Fe-NPc, except that the edges of the Fe-NPc islands appear less straight at higher amounts of Fe.

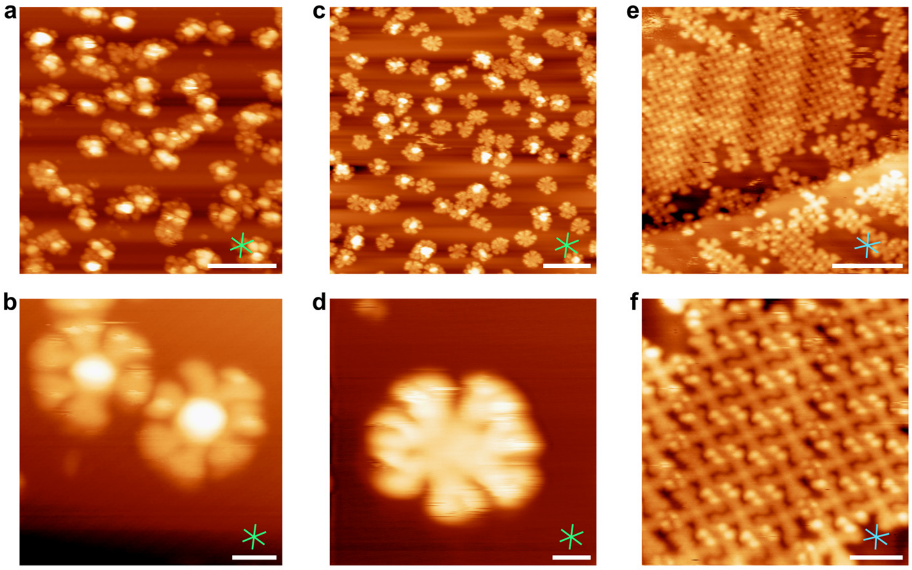

Figure S3. (a, c) Large-scale image of a PND/Fe/Ag(111) sample with an over-stoichiometric amount of iron (≈5.8 Fe per 4 monomers; cf. Figure 3: ≈0.7 Fe per 4 monomers) deposited at 300 K and annealed to 500 K, respectively. (b, d) Close-up images corresponding to (a) and (c), respectively. (e) Large-scale image of a PND/Fe/Au(111) sample with a larger sub-stoichiometric amount of Fe (≈0.8 Fe per 4 monomers; cf. Figure 6: ≈0.3 Fe per 4 monomers), after annealing to 500 K. (f) Close-up image of the island in (e), where individual regular Fe-NPc molecules can be seen. Bottom right: crystal orientation as determined in Figure S9. Scale bars: (a, c, e) 10 nm; (b, f) 3 nm; (d) 1 nm. Tunneling parameters: (a) U = -1.85 V, I = -0.11 nA; (b) U = -1.54 V, I = -0.11 nA; (c) U = -1.90 V, I = -0.13 nA; (d) U = -3.11 V, I = -0.16 nA; (e) U = -2.43 V, I = -0.14 nA; (f) U = -1.63 V, I = -0.13 nA.

2.4. Further Data on the Polycyanine and Ag-NPc Formation of PND

In a first attempt to obtain polycyanine chains, we annealed a PND multilayer (>18 ML; no substrate signals observable in XPS) on Ag(111) to 450 K. Subsequent STM measurements (Figure S4a) reveal no ordered structures. We believe that the obtained residual coverage (1.2 ML, according to XPS) is due to cross-linking of the organic molecules in the multilayer caused by X-rays and secondary electrons during the XPS measurements of the multilayer after deposition (Figure S4e left). Therefore, for the next preparation, the data of which are shown and discussed in the manuscript (cf. Figure 4), no XPS was measured before annealing. In this case, the coverage was determined by a quartz crystal microbalance.

Figure S4b-d show a close-up of the polycyanine chain that was obtained by annealing a PND multilayer that was not exposed to X-rays to 470 K. The same image is shown with three different overlays of hypothetical structures: the proposed polycyanine chain (Figure S4b) shows excellent agreement even for large numbers of repeat units; a coordination network of intact monomers with Ag adatoms and kinked bond angles (Figures S4c) fits similarly well along the chain, but is too large perpendicular to the chain direction; a metal-organic equivalent of the polycyanine chain with incorporated Ag atoms fits well in chain width, but is too long in the chain direction, as shown by the clear deviations already after few repeat units. This comparison provides further evidence for the formation of the covalent polycyanine chain.

Complementary XPS measurements (Figure S4e) support these findings. In the C 1s range of the PND multilayer XPS spectrum (Figure S4e, left), we find a large peak with a clearly separated shoulder to higher binding energies as discussed in detail for the sub-monolayers (Figure 5 in the manuscript). Upon annealing the intensity decreases significantly and a new peak appears at a lower binding energy.

XPS spectra taken after annealing the multilayer that was not exposed to X-rays to 450 K (Figure S4e right), we see a asymmetric peak. The asymmetry is attributed to the co-existence of reacted species and residual monomer molecules (as can be seen in Figure 4a). Upon further annealing to 470 K, the coverage decreases further (450 K: 0.51 ML; 470 K: 0.39 ML) and the peak becomes more symmetric. This is in line with the STM data, which show less monomers, while the chains and tetramers are the majority species. Further annealing to 550 K only leads to minor desorption (0.36 ML), supporting our observation of conversion of the chains into cyclic tetramers instead of decomposition and desorption.

To further identify the islands seen after annealing to 550 K we took close-up images of an islands (Figure S4f,g), where cyclic tetramers can clearly be identified. The molecules show bright central protrusions and are attributed to intact Ag-NPc in the Ag-up configuration.

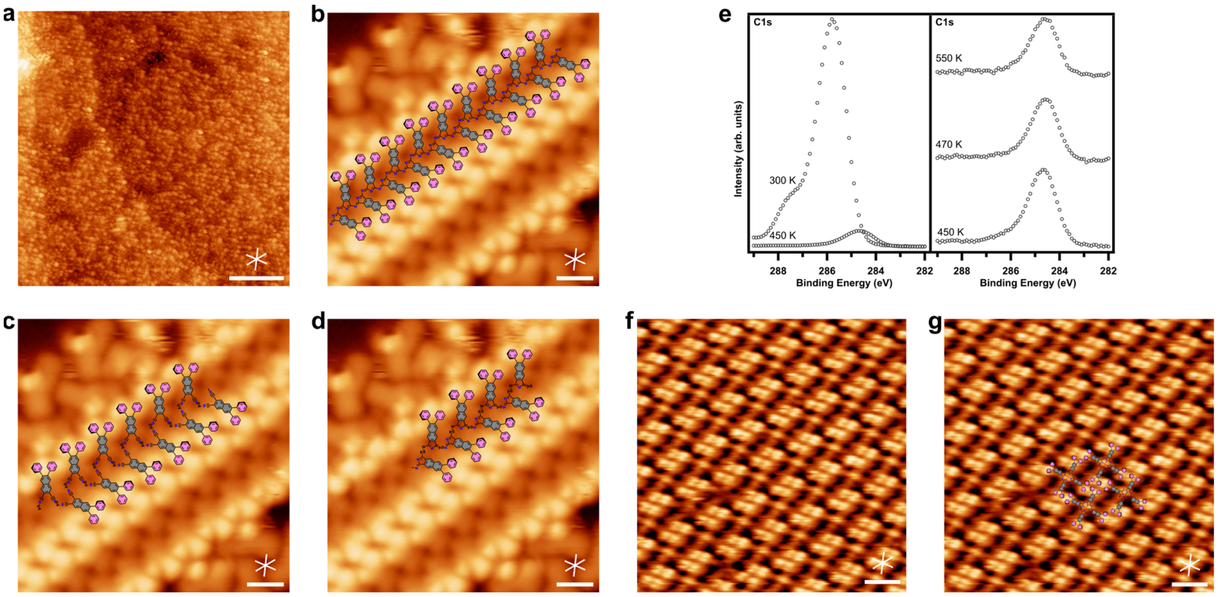

Figure S4. (a) STM image of a PND multilayer sample (>18 ML; no substrate signal visible in XPS) after annealing to 450 K (1.2 ML). This multilayer was exposed to X-ray radiation prior to the annealing. (b-d) Polycyanine chain obtained by annealing a PND multilayer on Ag(111) without prior X-ray exposure (cf. Figure 4) with different hypothetical molecular overlays, as is discussed in the text. (e) XPS spectra of the C 1s range for both PND multilayer experiments with (left) and without (right) XPS measurement at 300 K before annealing to 450 K (coverage right: 450 K: 0.51 ML; 470 K: 0.39 ML; 550 K: 0.36 ML). (f, g) Close-up STM image of an island as in Figure 4o featuring tetramers without and with molecular overlay, respectively. Scale bars: (a) 20 nm; (b-d) 1 nm; (f, g) 2 nm. Tunneling parameters: (a) U = 2.02 V, I = 0.14 nA; (b-d) U = -1.40 V, I = -0.24 nA; (f, g) U = -3.31 V, I = -0.13 nA.

2.5. Additional C 1s XPS Spectra

In Figure 5 in the manuscript, only a selection of XPS spectra is shown for the sake of clarity. However, taking a closer look at further C 1s spectra in Figure S5 provides additional insight. Already upon deposition of Fe onto PND on both surfaces, the peak shape changes substantially. Especially the shoulder towards higher binding energies decreases, which is probably a consequence of the coordination of the nitrile groups to iron atoms. However, since there is a sub-stochiometric amount of iron (cf. Figure S3 and S8), uncoordinated nitrile groups are present as well. After annealing to 500 K, the coverage is reduced in all cases. For PND, this is easily understood taking the amount of iron into consideration. At 500 K there is roughly 1 Fe left for each tetramer, while the residual monomers desorb. For NND, the explanation might be similar, i.e., the residual amount may depend on the Ag adatom concentration.

Further annealing at 630 K slightly decreases the coverage in the cases of PND/Fe/Au(111) (Figure S5a) and NND/Ag(111) (Figure S5c). For PND/Fe/Au(111), we attribute this to desorption of unreacted monomers (cf. Section 2.8).

In addition, we observe small changes in the peak width upon the annealing from 500 K to 630 K (increase of FWHM: Figure S5a, 0.98 eV to 1.05 eV; Figure S5b, 1.07 eV to 1.18 eV; Figure S5c, 1.21 eV to 1.30 eV), which are attributed to increasing inhomogeneity due to the coexistence of individual flat M-NPc molecules and already interconnected irregular networks as shown for NND (cf. Figure S1a and b).

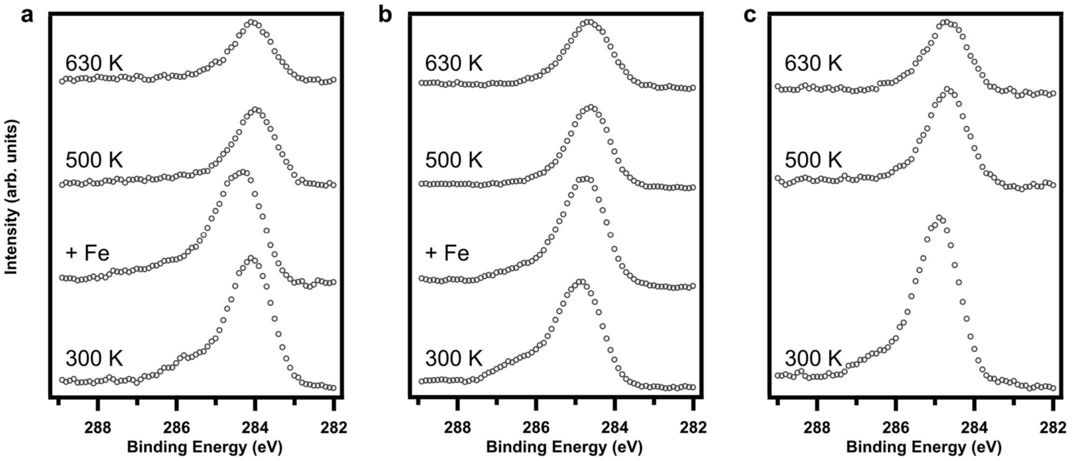

Figure S5. XP Spectra of the C 1s regime for (a) PND/Fe/Au(111), (b) PND/Fe/Ag(111) and (c) NND/Ag(111). Shown are for each the spectra taken after deposition of the precursors at 300 K, after reaction at 500 K and further annealing to 630 K. For the smaller PND additionally the co-adsorbate with iron is shown. Coverages: (a) 300 K: 0.67 ML, 500 K: 0.34 ML, 630 K: 0.26 ML; (b) 300 K: 0.52 ML, 500 K: 0.33 ML, 630 K: 0.30 ML; (c) 300 K: 0.76 ML, 500 K: 0.40 ML, 630 K: 0.30 ML.

2.6. Desorption of PND from the Au(111) Surface

After annealing a sub-monolayer (0.61 ML) of PND on Au(111) to 500 K, the STM image (Figure S6a) show no longer any regular structures, but only small amounts or irregular residues. The absence of any regular structures suggests that there is no reaction of PND to Au-NPc or any chain-type structure. In line with this, the C 1s XPS (Figure S6b) shows a significantly decreased intensity (to 0.12 ML) and slight changes to the peak shape.

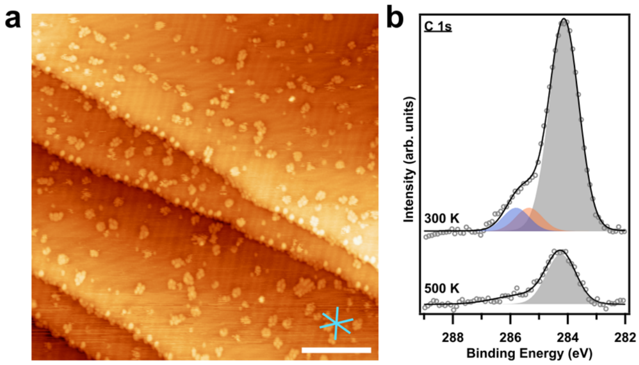

Figure S6. (a) Large scale image of 0.61 ML PND on Au(111) annealed to 500 K without co-adsorbed metal. (b) XP spectra showing the desorption of molecules upon annealing. Scale bar: (a) 30 nm; Tunneling parameters: (a) U = -2.59 V, I = -0.10 nA; Bottom right: crystal orientation as determined in Figure S9.

2.7. Reactivity of NND on Au(111) and Formation of Au-NPc

As discussed in the main text, the choice of substrate (Ag(111) or Au(111)) had only minor influence on the on-surface reactivity of PND with Fe. The only significant difference is in the shape of the island of the final Fe-NPc product.

More substantial differences were observed for NND on the two surfaces regarding the formation of metallonaphthalocyanines. For comparison with the Ag(111) case discussed in the main text, we prepared a sub-monolayer of NND on Au(111) at 300 K. In corresponding STM images (Figure S7a,b), regular arrangements can be seen, however, due to the limited resolution, no exact molecular structure can be deduced. Annealing to 500 K results neither in desorption nor in an obvious reaction of the molecules (Figure S7c,d). Only by further increasing the annealing temperatures to 550 K and, thereafter, to 630 K, a small fraction of cyclic tetramers can be seen (Figure S7e,f and S7g,h, respectively). Some appear with a bright center, while others have a dark center, similarly as observed for the Ag complexes. It is concluded that the formation of Au-NPc on Au(111) is much less efficient than the formation of Ag-NPc on Ag(111), presumably due to the lower reactivity of Au.

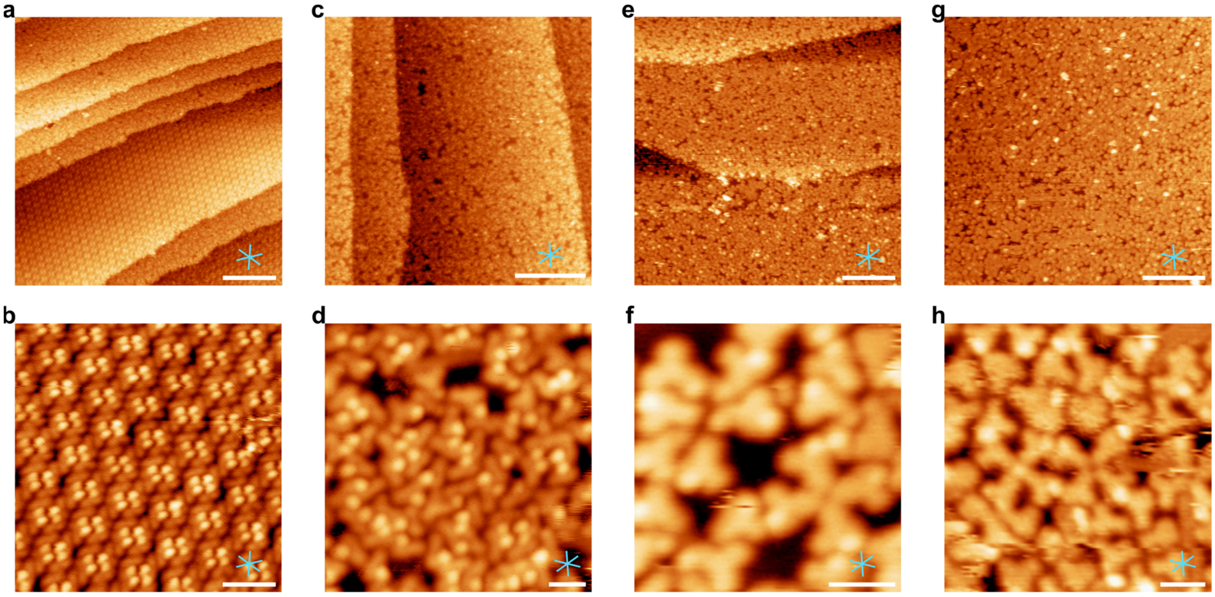

Figure S7. (a, c, e, g) Large-scale images of a NND sample on Au(111) annealed to increasing temperatures: 300 K, 500 K, 550 K, 600 K. (b, d, f, h) Corresponding close-up images. (b) Upon deposition at 300 K some regular structures in a quasi-hexagonal lattice are observed. (d) Annealing to 500 K leads to random ordering of precursor shaped features. (f) Further annealing to 550 K leads to rarely encountered quasi fourfold symmetric features we attribute to coordination to a gold adatom. (h) Annealing to 600 K shows two phthalocyanine shaped features next to each other. However, in the center of the left one a bright protrusion is seen, which indicates the presence of a metal center. Scale bars: (a, c, e, g) 20 nm; (b) 4 nm; (d, f, h) 2 nm. Tunneling parameters: (a) U = 2.43 V, I = 0.08 nA; (b) U = 2.43 V, I = 0.12 nA; (c) U = 1.79 V, I = 0.09 nA; (d) U = 1.79 V, I = 0.10 nA (e) U = -2.93 V, I = -0.10 nA; (f) U = -3.52 V, I = -0.14 nA; (g) U = -3.11 V, I = -0.17 nA; (h) U = -3.02 V, I = -0.14 nA. Bottom right: crystal orientation as determined in Figure S9.

2.8. Further STM Data of PND on Au(111)

Beside the structures shown in Figure 6 of the main text, PND on Au(111) features some less understood and, in this study, less thoroughly explored structural motifs. Figure S8 shows selected STM data and structural models. Figure S8a, b and c display the two observed self-assembled structures of PND on Au(111) as seen in the overview in Figure 6b and a proposed structural model (Figure S8d). Figure S8b shows a close-up of the tetramer-like motif, which is shown but not discussed in detail in the main text, with overlaid chemical models. Figure S8e and Figure S8g show STM images of the coordination network formed after adsorption of sub-stoichiometric amounts of Fe onto the pristine PND layer on Au(111) held at 300 K, before reaction at 500 K. In the overview image, one can see two different island motifs, closed-packed (Figure S8f) and row-like (Figure S8h). These two phases may result from different local molecule-to-iron ratios. However, while the close-up STM image (Figure S8g) shows a proposed chemical model, we were not able to resolve individual iron atoms and thus cannot inquire further.

After annealing this Fe/PND/Au(111) sample to 500 K, we observed the well-ordered Fe-NPc islands and another phase, both of which appear in the overview STM image in Figure 6d. Figure S8i shows a close-up of the other phase. The structure of this phase seems to closely resemble that of the iron-PND coordination network of the unreacted Fe/PND phase in Figure S8g. Therefore, we directly compare both phases (Figure S8j: mirrored and rotated cut-out of Figure S8i; Figure S8k: close-up image of the row-like network in Figure S8g). Despite the limited resolution, it can be concluded that Figure S8i shows a mirror domain of the iron-coordinated network shown in S8h. This implies that the reaction is not complete at 500 K, but results in the coexistence of the Fe-NPc product and the iron-PND coordination network. Figure S8l shows the STM image of a sample with higher iron-to-PND ratio (cf. Section 2.3) annealed to 550 K. The image shows immobile cyclic tetramers, which predominantly adsorb near the elbow sites. The other parts of the surface appear blurry, but the blurry features follow the herringbone reconstruction of the Au(111) surface. The molecular backbone appears with uniform brightness without pronounced protrusions, suggesting that the ligands have been flattened due to dehydrogenation reactions (as discussed in the main text). The flattening results in increased mobility resulting is the mostly blurry image. Only those of the flattened molecule which have engaged in intermolecular linking are sufficiently immobile for resolvable imaging under the applied experimental conditions.

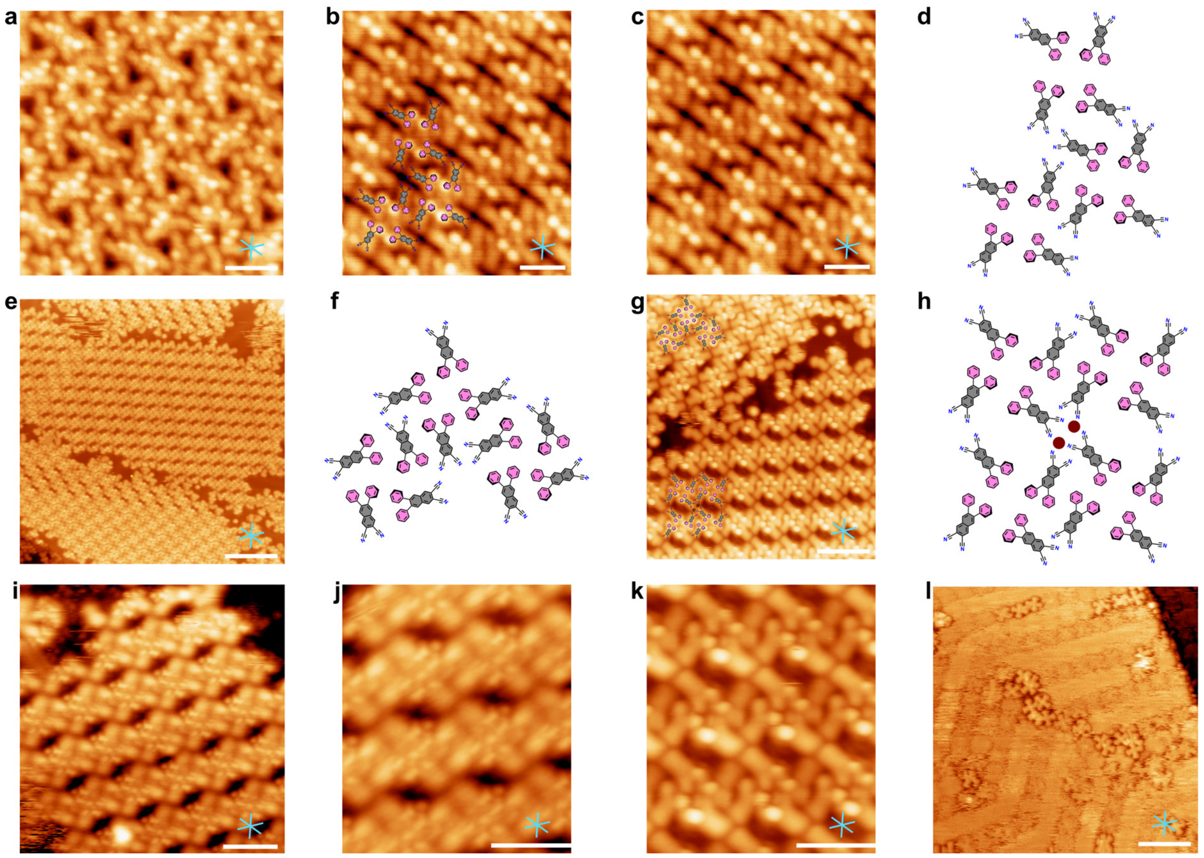

Figure S8. (a) STM image of PND on Au(111) as deposited at 300 K, showing a larger area compared to Figure 6c. (b,c) Close-up STM images of the second PND structure formed of this sample with (b) and without (c) molecular overlay. An enlarged version of the overlaid molecular model is shown again in (d). (e) Overview STM image taken after deposition of a sub-stoichiometric amount of iron onto the PND layer. (f-h) Proposed structures as seen in the close-up image (g) without (f) and with (h) incorporation of iron atoms. (i) STM image of the row-like assembly of tetramer-type structures observed after annealing to 500 K. (j) Mirrored and rotated cut-out of i) in contrast to the coordinated network from g). (l) Overview STM image featuring very mobile species upon annealing a sub-monolayer PND with increased amounts of iron to 550 K (cf. Figure S3e and f). Scale bars: (a, I, j, k) 3 nm; (b, c) 2 nm; (e, l) 10 nm; (g) 4 nm. Tunneling parameters: (a) U = 2.02 V, I = 0.09 nA; (b, c) U = -1.00 V, I = -0.18 nA; (e) U = -2.59 V, I = -0.23 nA; (g) U = -2.59 V, I = -0.16 nA (i, j) U = -0.63 V, I = -0.10 nA; (k) U = -2.59 V, I = -0.16 nA; (l) U = 2.51 V, I = 0.11 nA. Bottom right: crystal orientation as determined in Figure S9.

2.9. Determination of the Crystal Orientation

Typically, we are not able to resolve the molecular overlayer and the substrate structure in the same image. Therefore, the substrate orientation was determined by the following approaches. Figure S9a shows an STM image of the Au(111) surface after desorption of PND with the herringbone reconstruction visible. The bright stripes of the reconstruction are oriented along the densely packed [11-2] direction (and equivalent directions). The blue lines thus show the orientation of the high symmetry directions. These orientations are used to indicate the substrate lattice for all samples prepared on Au(111). For Ag(111), a separately recorded image of the surface (Figure S9b) was used. Figure S9c shows a cut-out of the cleanly visible substrate and the determined orientation by green lines. For the PND experiments with multilayer coverage the usage of a different Ag(111) crystal was necessary. Figure S9d shows the corresponding substrate orientation for this crystal.

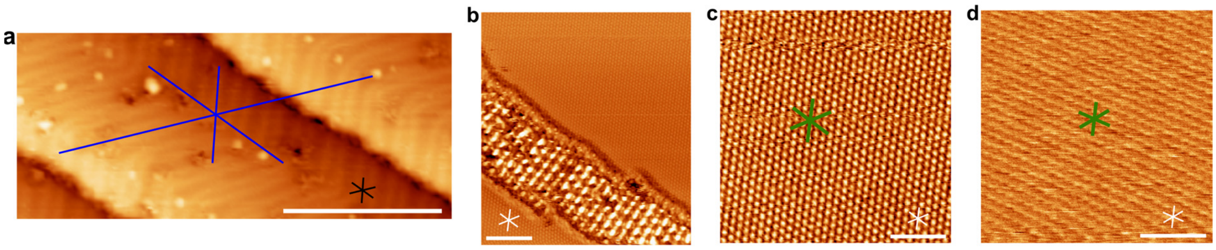

Figure S9. (a) STM image taken after desorbing PND from Au(111) (c.f. Figure S6). The herringbone reconstruction was used to determine the crystal orientation. (b) STM image of the used Ag(111) single crystal from a different preparation with atomically resolved substrate. (c) Cutout from (b) with determined crystal orientation d) STM image of the Ag(111) crystal used for the multilayer experiments of PND. Scale bars: (a) 30 nm; (b) 4 nm; (c, d) 2 nm. Tunneling parameters: (a) *U* = -2.51 V, *I* = -0.10 nA; (b, c) *U* = -0.29 V, *I* = -0.14 nA; (d) *U* = -0.78 V, *I* = -0.18 nA.

2.10. Size Adjustments of Molecular Overlays

To make the molecular overlays for the STM data in Figures 2, 3, 4 and 6, it was necessary to scale the skeletal formulas. This was done according to values obtained by MM2 optimizations in Chem3D by PerkinElmer Informatics for atomic distances in the planar parts of the molecules (shown in Figure S10). This does neither include deformations due to the surface interaction nor the influence of the local density of states for the STM contrast. This and individual drift compensation make the molecular overlays only a rough estimate.

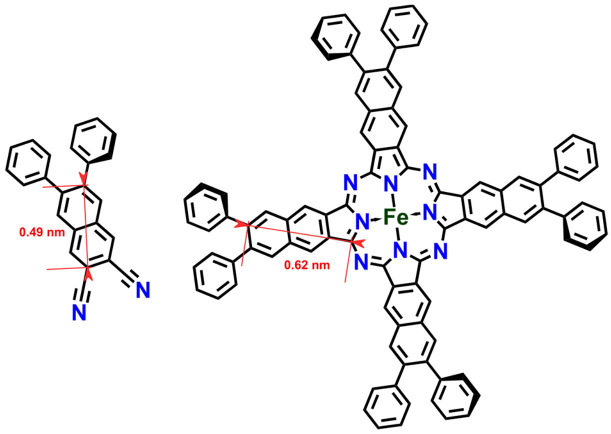

Figure S10. Used distances to scale the molecular structures shown in the STM images. The values were obtained by MM2 geometry optimizations in Chem3D 21 by PerkinElmer Informatics.
